# Supplementary material for: Hippocampal-cingulate dynamics in the human brain link reinforcement-learning and memory
Source: bioRxiv. 2026 Sep 12:2026.09.10.750374. Preprint. [Version 1] doi: 10.64898/2026.09.10.750374 (PMC13622676; doi:10.64898/2026.09.10.750374)
Supplement: Supplementary file 1 [file NIHPP2026.09.10.750374V1-supplement-1.pdf]

bioRxiv preprint doi: https://doi.org/10.64898/2026.09.10.750374; this version posted September 12, 2026. The copyright holder for this preprint (which was not certified by peer review) is the author/funder, who has granted bioRxiv a license to display the preprint in perpetuity. It is made available under a CC-BY 4.0 International license.

**model**	**parameters**
WSLS	ϵ
**RW**	α, β
RWF	α, β
RWA	d, β
VKF	ω, β
Bayes	p_{switch}, β

Table 1: Models, along with free parameters, are utilized for model comparison. The bold row denotes the winning model.

bioRxiv preprint doi: https://doi.org/10.64898/2026.09.10.750374; this version posted September 12, 2026. The copyright holder for this preprint (which was not certified by peer review) is the author/funder, who has granted bioRxiv a license to display the preprint in perpetuity. It is made available under a CC-BY 4.0 International license.

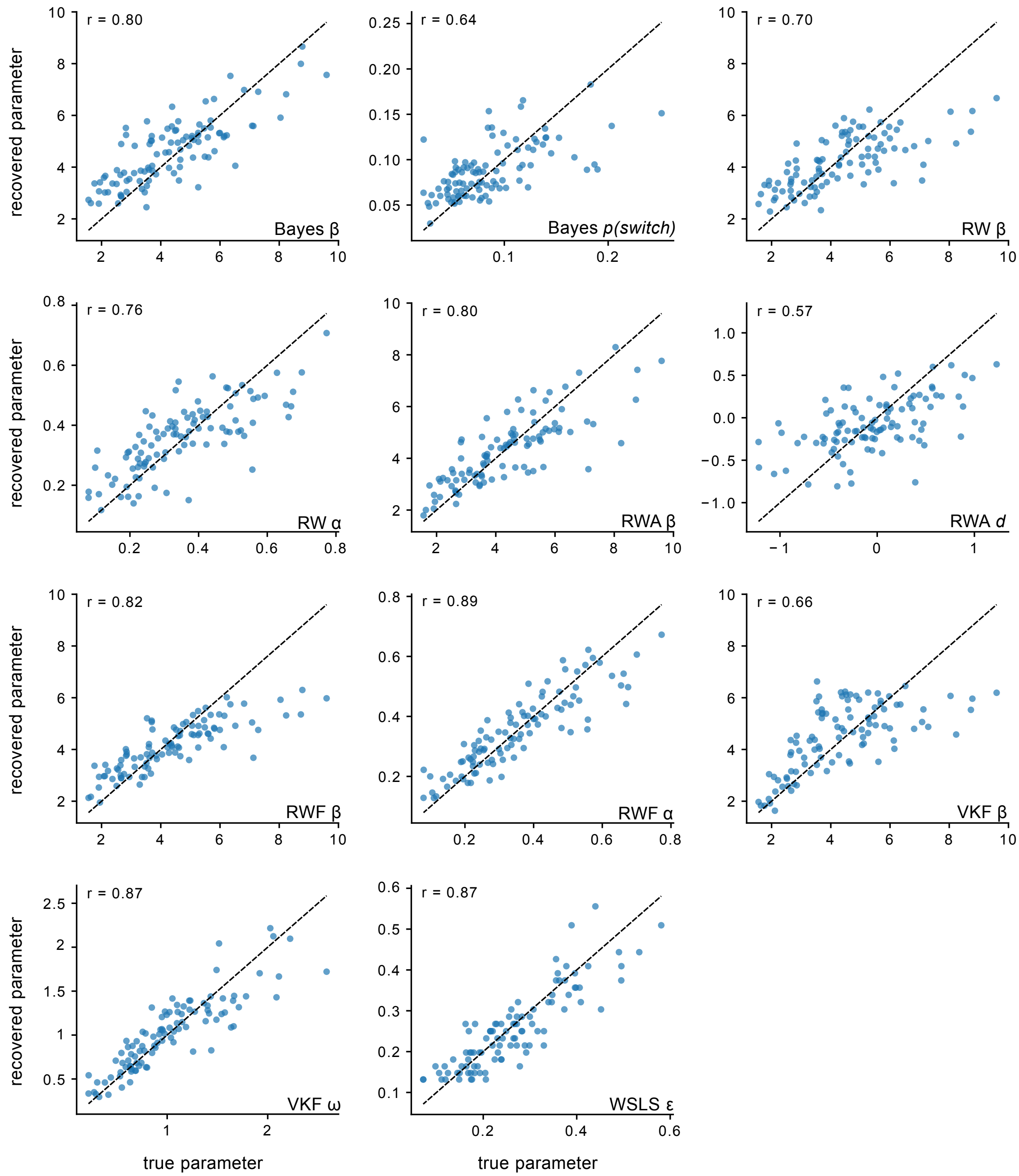

Figure S1: **Parameter recovery for candidate reinforcement-learning models.** Recovered vs. true parameter values from equal-design parameter recovery (N = 100 simulated subjects × 80 trials per model, matching the empirical task design), re-estimated via hierarchical expectation-maximization using the same production settings applied to real data. Each panel corresponds to one free parameter of one candidate model: Bayes (fixed-reward) inverse temperature (β) and switch probability (p(switch)); Rescorla–Wagner (RW) learning rate (α) and inverse temperature (β); valence-asymmetric RW (RWA) inverse temperature (β) and asymmetry (d); fictive RW (RWF) learning rate (α) and inverse temperature (β); volatility Kalman filter (VKF) inverse temperature (β) and volatility-learning rate (ω); and win-stay-lose-shift (WSLS) lapse rate (ε). Dashed lines indicate the identity line (perfect recovery); Pearson's r between true and recovered values is reported in the top-left of each panel.

bioRxiv preprint doi: https://doi.org/10.64898/2026.09.10.750374; this version posted September 12, 2026. The copyright holder for this preprint (which was not certified by peer review) is the author/funder, who has granted bioRxiv a license to display the preprint in perpetuity. It is made available under a CC-BY 4.0 International license.

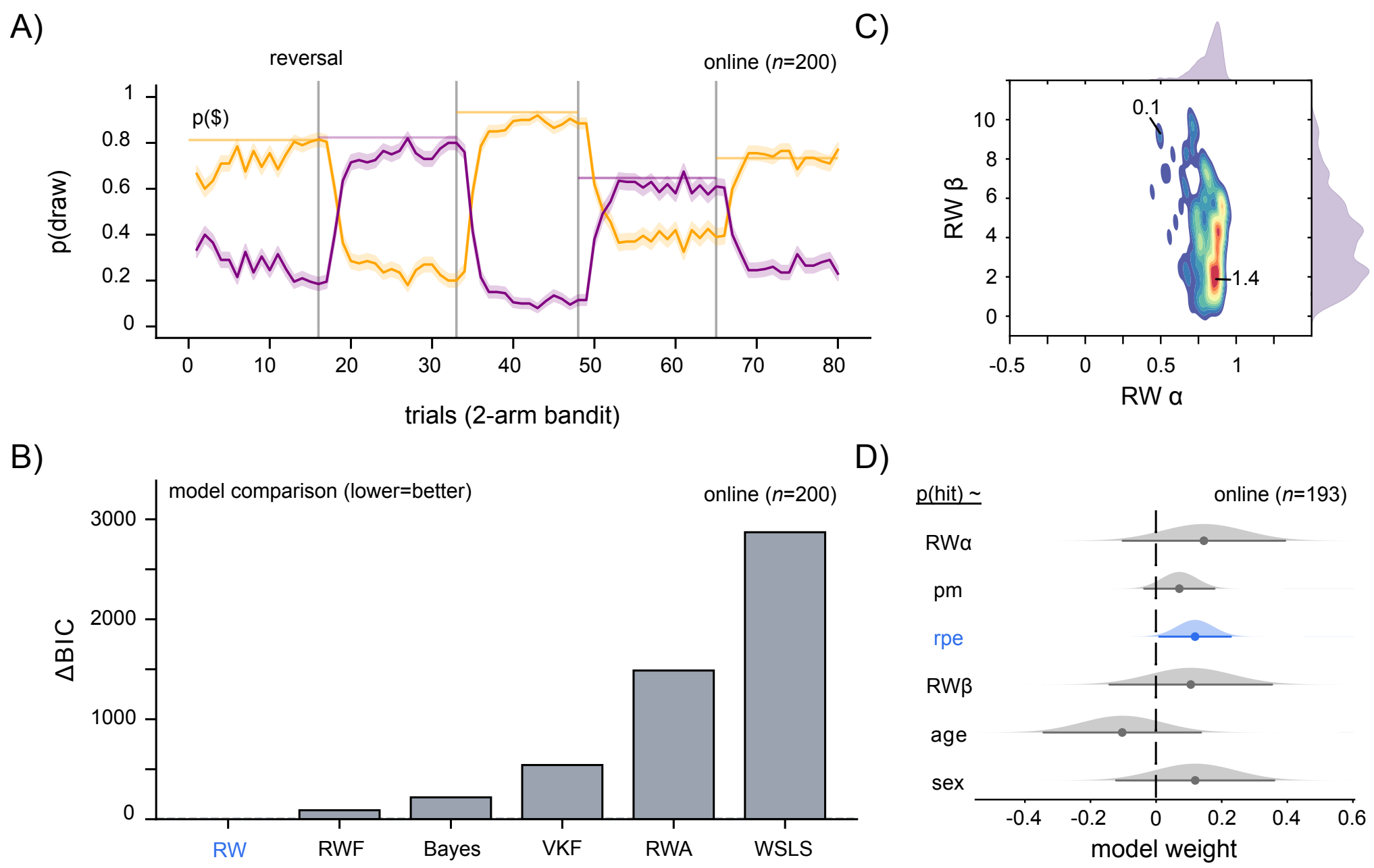

Figure S2: **Healthy prolific cohort behavioral validation.** A) Probability of drawing from the male (orange) or female (purple) decks as a function of trial and reward block (mean reversal trial indicated by vertical lines). Horizontal lines denote probability of reward for the more rewarding deck on each block. Shaded lines denote a 95% confidence interval. B) Model performance, specified by the integrated BIC across six candidate reinforcement-learning models fit hierarchically to subjects' choice sequences on the 2-arm bandit task. Bar height represents ΔBIC, each model's integrated BIC relative to the best-fitting model in the set (best = 0); lower is better. C) Joint and marginal distributions of parameter estimates from the RW model, depicting the learning rate (α) and inverse temperature (β). Warm colors indicate higher density. D) Sampling distributions for the fixed effects in a binomial mixed-effects model examining how trial-level reward prediction error (rpe), image memorability (pm), subject-level Rescorla–Wagner parameters (learning rate α, inverse temperature β), age, and sex influence recognition memory success in the online cohort (n=193). Each curve is a normal approximation to the coefficient's sampling distribution, and the horizontal bar spans the 95% Wald confidence interval. The dashed vertical line indicates a coefficient of 0. Distributions whose 95% CI excludes 0 are shaded blue, indicating a statistically reliable effect, while those whose CI includes 0 are shaded gray.

bioRxiv preprint doi: https://doi.org/10.64898/2026.09.10.750374; this version posted September 12, 2026. The copyright holder for this preprint (which was not certified by peer review) is the author/funder, who has granted bioRxiv a license to display the preprint in perpetuity. It is made available under a CC-BY 4.0 International license.

Table 2: Participants and number of electrodes per region

participant	# of electrodes per region					
	OFC	ACC	dmPFC	dlPFC	HPC	AMY
1	5	7	10	0	3	3
2	6	5	7	5	9	5
3	4	6	8	2	2	2
4	5	5	7	2	4	5
5	4	15	10	8	6	2
6	0	3	7	6	4	3
7	9	7	17	5	6	6
8	13	11	5	2	5	5
9	14	10	5	0	8	1
10	11	16	22	4	7	4
11	4	9	5	2	4	2
12	11	12	13	7	3	7
13	16	4	7	16	6	3
14	0	0	0	0	4	4
15	7	7	8	0	2	11
16	0	0	0	0	4	6
17	0	0	0	0	9	2
18	14	0	6	0	6	2
19	11	3	7	2	4	3
**Total**	**134**	**120**	**144**	**61**	**96**	**76**

bioRxiv preprint doi: https://doi.org/10.64898/2026.09.10.750374; this version posted September 12, 2026. The copyright holder for this preprint (which was not certified by peer review) is the author/funder, who has granted bioRxiv a license to display the preprint in perpetuity. It is made available under a CC-BY 4.0 International license.

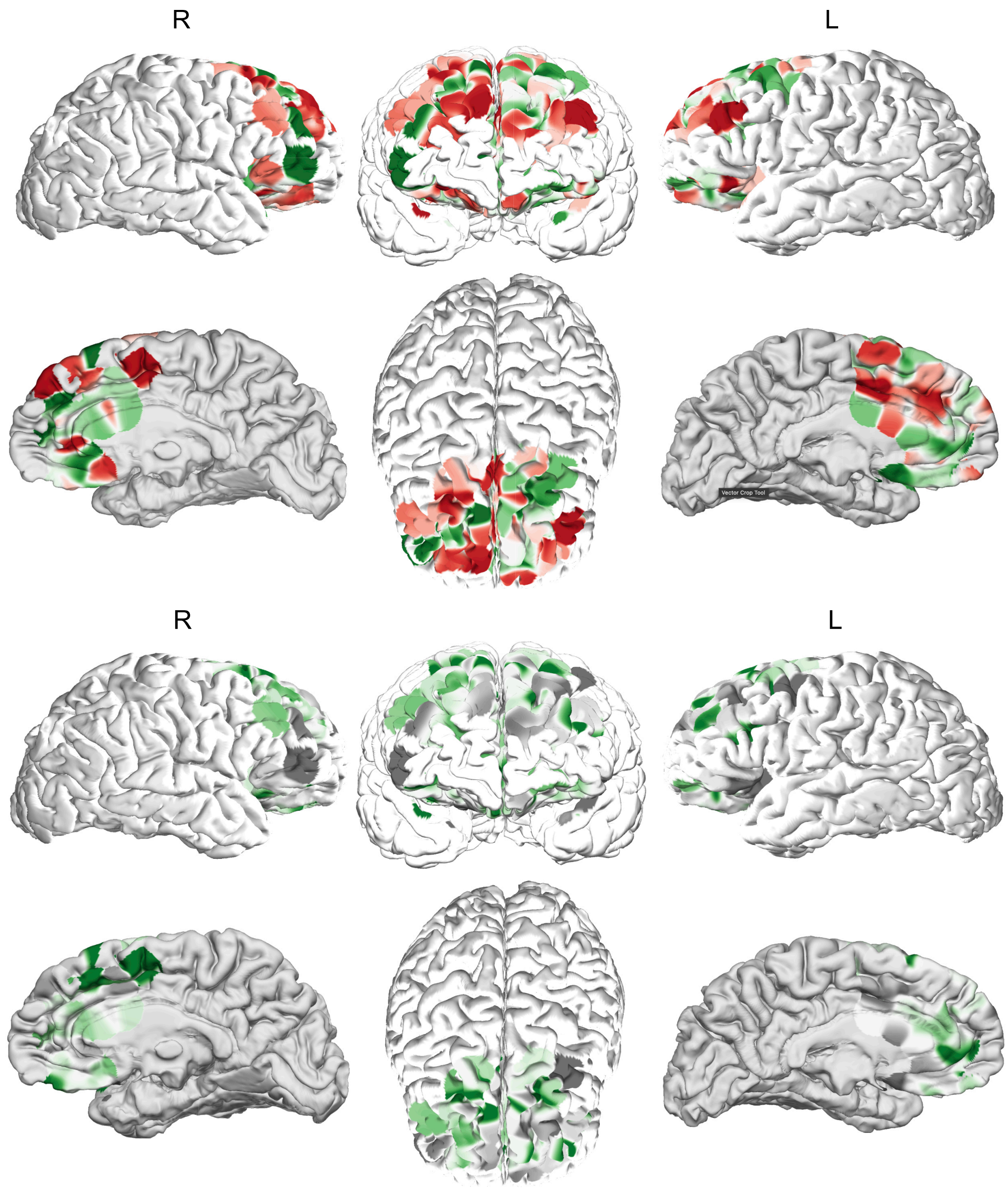

Figure S3: **Prefrontal activation patterns for positive, negative, and neutral RPE encoding during feedback.** Prefrontal activation patterns computed by multiplying the model weight vector by the feature covariance matrix, yielding a pattern that reflects how each electrode covaries with positive RPE compared to negative RPE (top) and neutral RPE (bottom). Timepoints for visualization were selected from the significant clusters for each individual ROI: OFC, ACC, dmPFC. Green denotes increased activation for positive RPEs, red denotes increased activation for negative RPEs (top), and gray denotes increased activation for neutral RPEs (bottom).

bioRxiv preprint doi: https://doi.org/10.64898/2026.09.10.750374; this version posted September 12, 2026. The copyright holder for this preprint (which was not certified by peer review) is the author/funder, who has granted bioRxiv a license to display the preprint in perpetuity. It is made available under a CC-BY 4.0 International license.

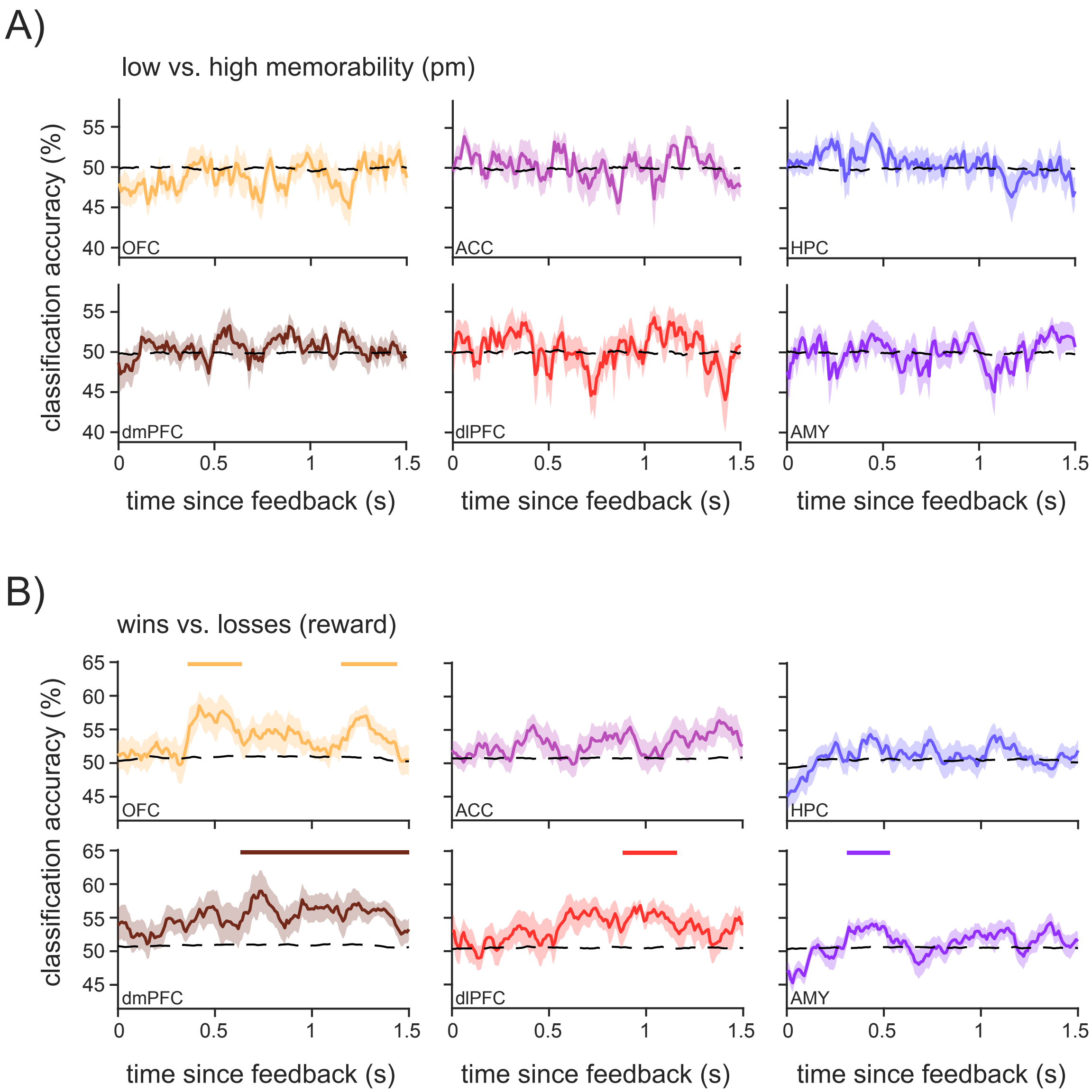

Figure S4: **Perceptual memorability cannot be decoded during feedback.** A) Cross-validated binary LDA classifier accuracy for memorability (high vs. low, median split) for each region of interest. The dotted line denotes the mean of the surrogate values. Solid lines denote significant time clusters computed using shuffled null data. Shaded lines indicate standard error across subjects. B) Cross-validated binary LDA classifier accuracy for reward outcome (wins vs. losses) for each region of interest. The dotted line denotes the mean of the surrogate values. Solid lines denote significant time clusters computed using shuffled null data. Shaded lines indicate standard error across subjects.

bioRxiv preprint doi: https://doi.org/10.64898/2026.09.10.750374; this version posted September 12, 2026. The copyright holder for this preprint (which was not certified by peer review) is the author/funder, who has granted bioRxiv a license to display the preprint in perpetuity. It is made available under a CC-BY 4.0 International license.

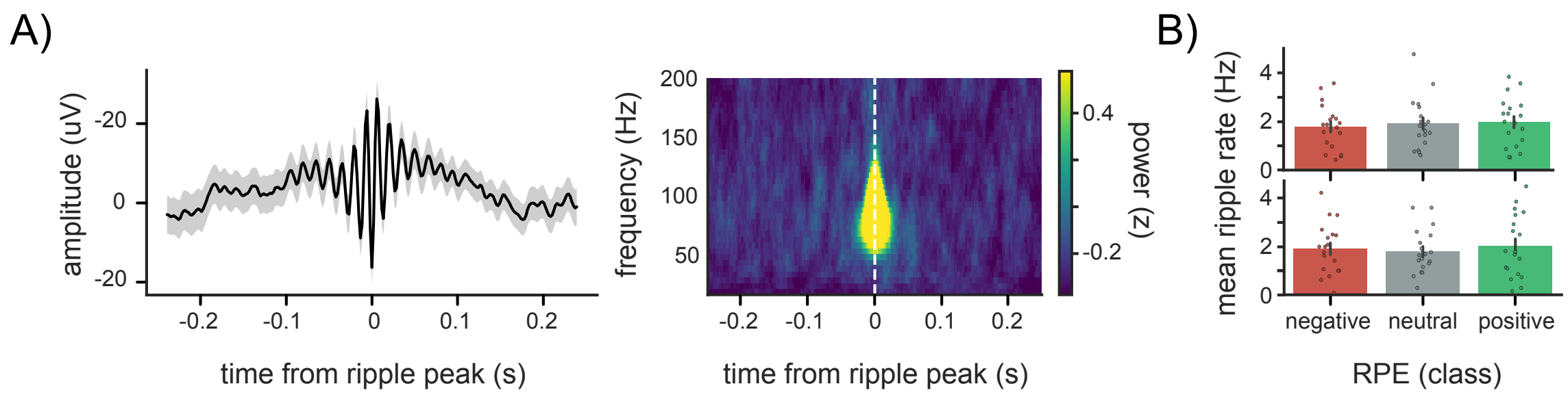

Figure S5: **Hippocampal ripple rate does not differ as a function of RPE.** A) Left: example raw LFP trace of grand-average ripple (one subject; 340 ripples) in the hippocampus. Solid line denotes the mean across ripples. Shaded line denotes standard error. Right: spectrogram of the detected ripples in the same example subject. Yellow denotes higher power. B) Comparison of ripple rates between feedback (top) and recognition cue periods (bottom). Bar heights denote the mean hippocampal ripple rate across subjects (dots). Error bars denote standard error.

bioRxiv preprint doi: https://doi.org/10.64898/2026.09.10.750374; this version posted September 12, 2026. The copyright holder for this preprint (which was not certified by peer review) is the author/funder, who has granted bioRxiv a license to display the preprint in perpetuity. It is made available under a CC-BY 4.0 International license.

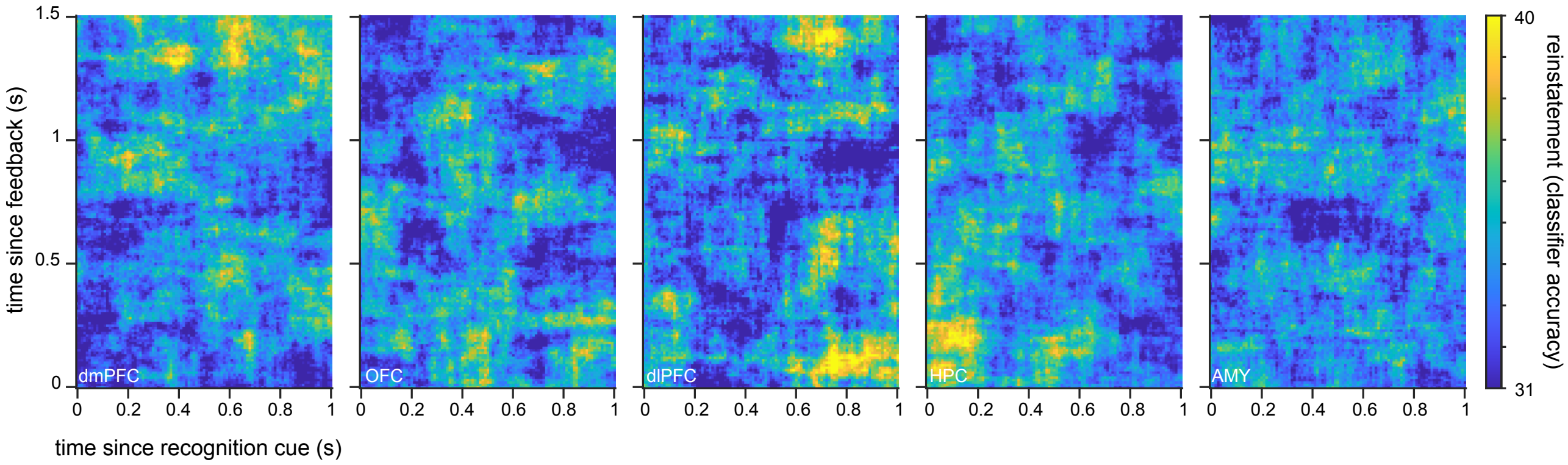

Figure S6: **Cross-decoding for other ROI.** Performance of cross-decoding classifier for RPE class trained on the feedback period (y-axis) and tested on the recognition cue period (x-axis), for all other regions of interest. Warmer colors indicate higher classifier performance.

bioRxiv preprint doi: https://doi.org/10.64898/2026.09.10.750374; this version posted September 12, 2026. The copyright holder for this preprint (which was not certified by peer review) is the author/funder, who has granted bioRxiv a license to display the preprint in perpetuity. It is made available under a CC-BY 4.0 International license.

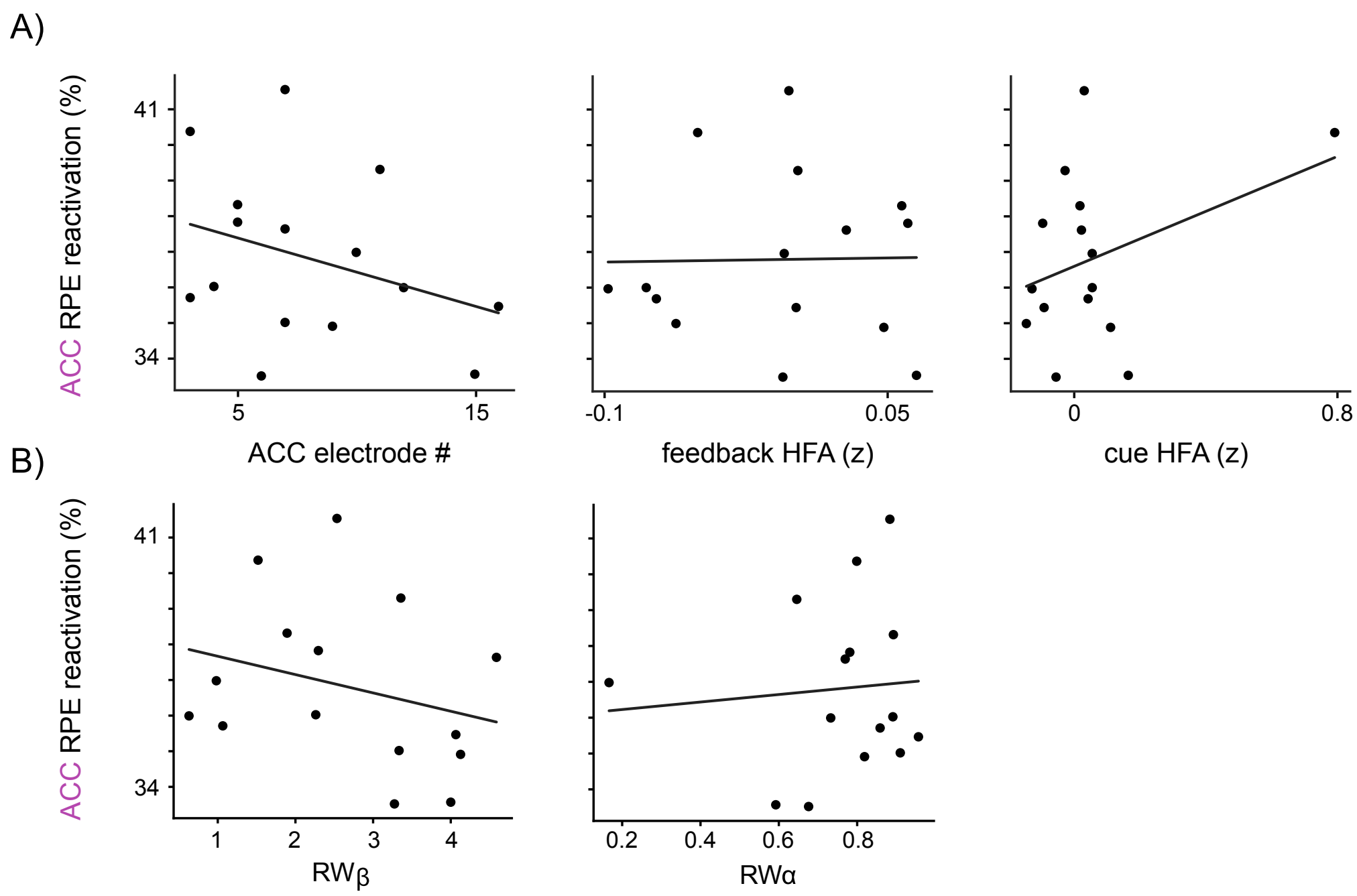

Figure S7: **ACC reinstatement not explained by subject-level covariates.** A) Scatter plots of the relationship between ACC reactivation in the significant cluster and: the number of ACC electrodes (left), overall ACC HFA during the feedback period (middle), and overall ACC HFA during the cue period (right). B) Scatter plots of the relationship between ACC reactivation in the significant cluster and: the learning rate (left), and inverse temperature (right).

bioRxiv preprint doi: https://doi.org/10.64898/2026.09.10.750374; this version posted September 12, 2026. The copyright holder for this preprint (which was not certified by peer review) is the author/funder, who has granted bioRxiv a license to display the preprint in perpetuity. It is made available under a CC-BY 4.0 International license.

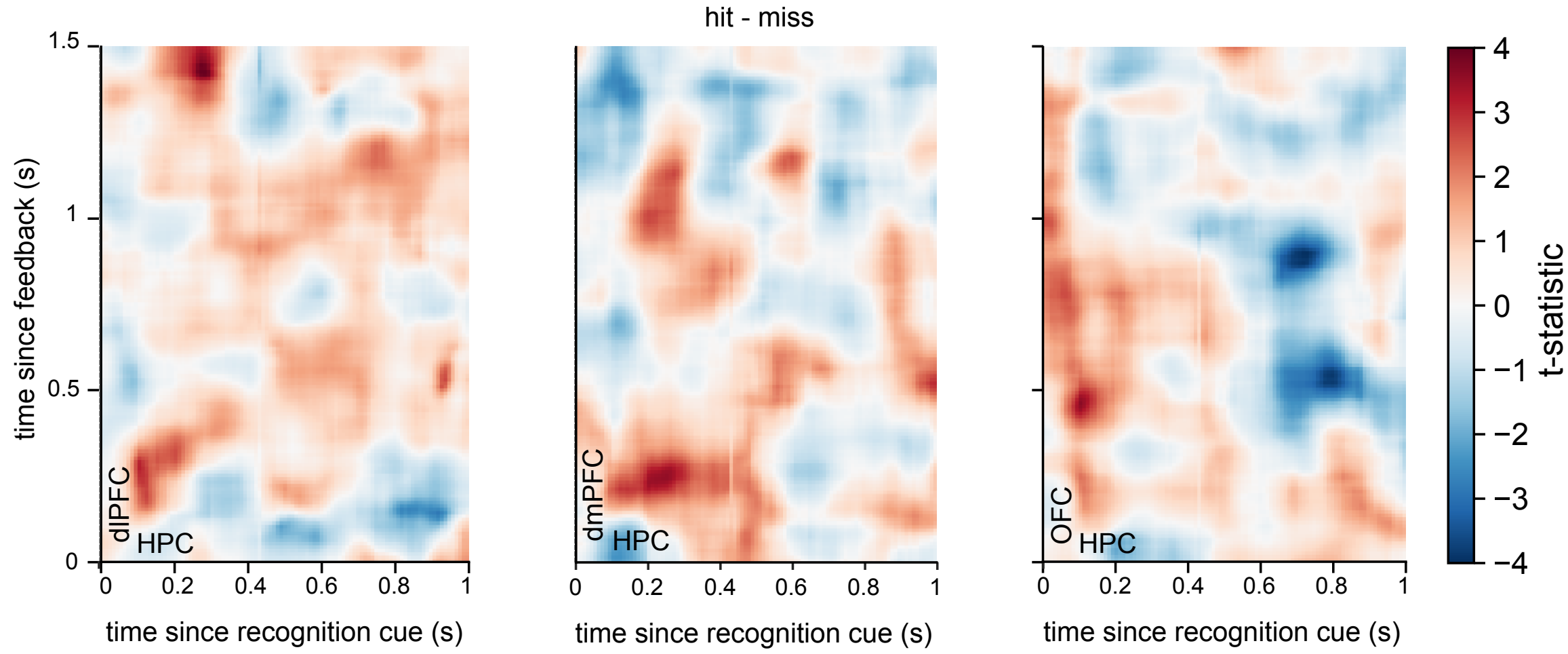

Figure S8: **Between-ROI RSA analyses.** Contrast heatmap of z-scored representational similarity (RSA) for all feedback and cue timepoints (hits vs. misses) for between-ROI analysis for dlPFC, dmPFC, and OFC feedback activity compared to HPC cue activity. Warmer colors denote higher RSA for hit trials compared to miss trials. Outline denotes significant cluster using permutation-cluster test.

bioRxiv preprint doi: https://doi.org/10.64898/2026.09.10.750374; this version posted September 12, 2026. The copyright holder for this preprint (which was not certified by peer review) is the author/funder, who has granted bioRxiv a license to display the preprint in perpetuity. It is made available under a CC-BY 4.0 International license.

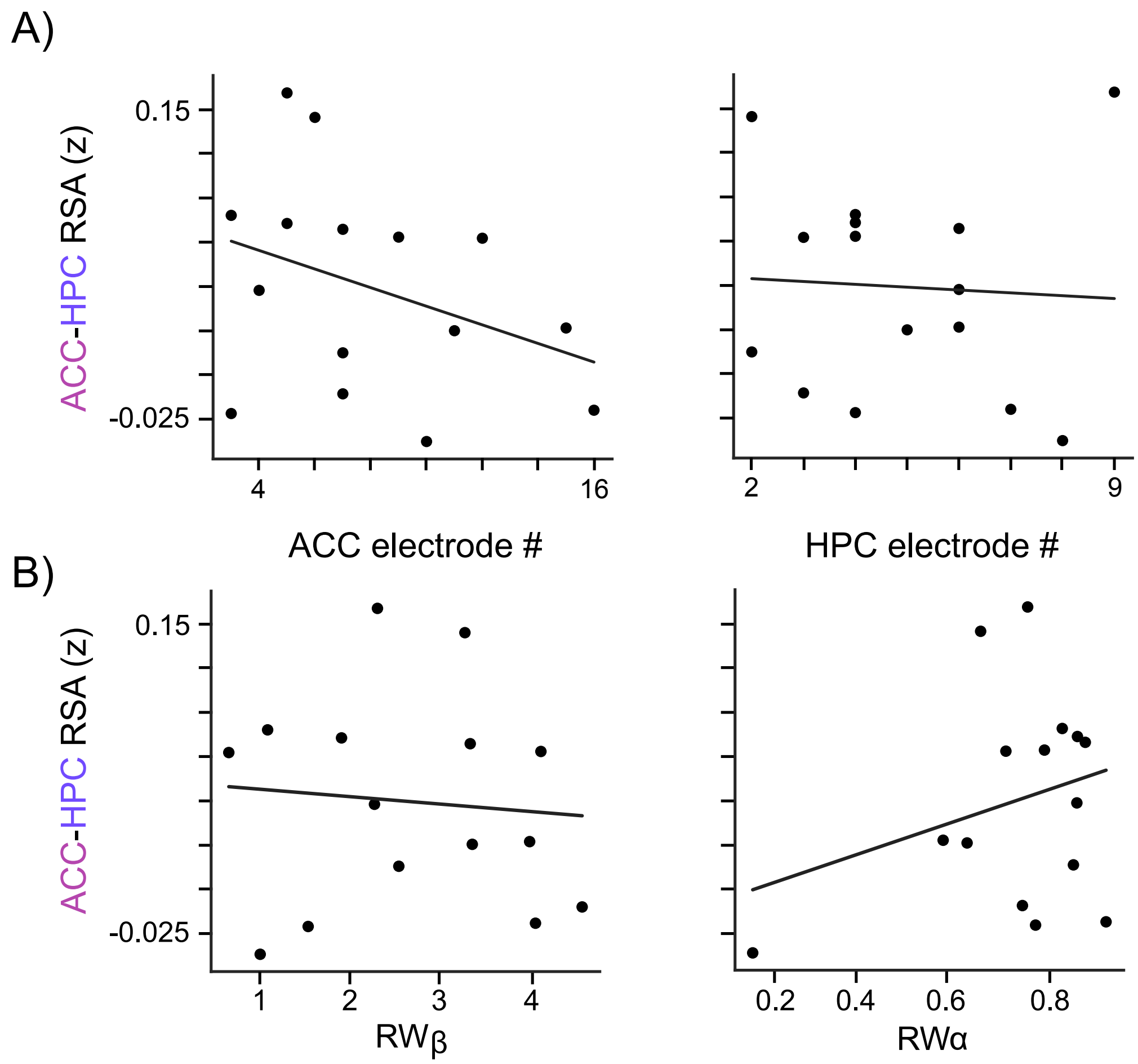

Figure S9: **HPC reinstatement of ACC feedback activity not explained by subject-level covariates.** A) Scatter plots of the relationship between ACC-HPC RSA and: the number of ACC electrodes (left), the number of HPC electrodes (right). B) Scatter plots of the relationship between ACC-HPC RSA and: the learning rate (left), and inverse temperature (right).

bioRxiv preprint doi: https://doi.org/10.64898/2026.09.10.750374; this version posted September 12, 2026. The copyright holder for this preprint (which was not certified by peer review) is the author/funder, who has granted bioRxiv a license to display the preprint in perpetuity. It is made available under a CC-BY 4.0 International license.

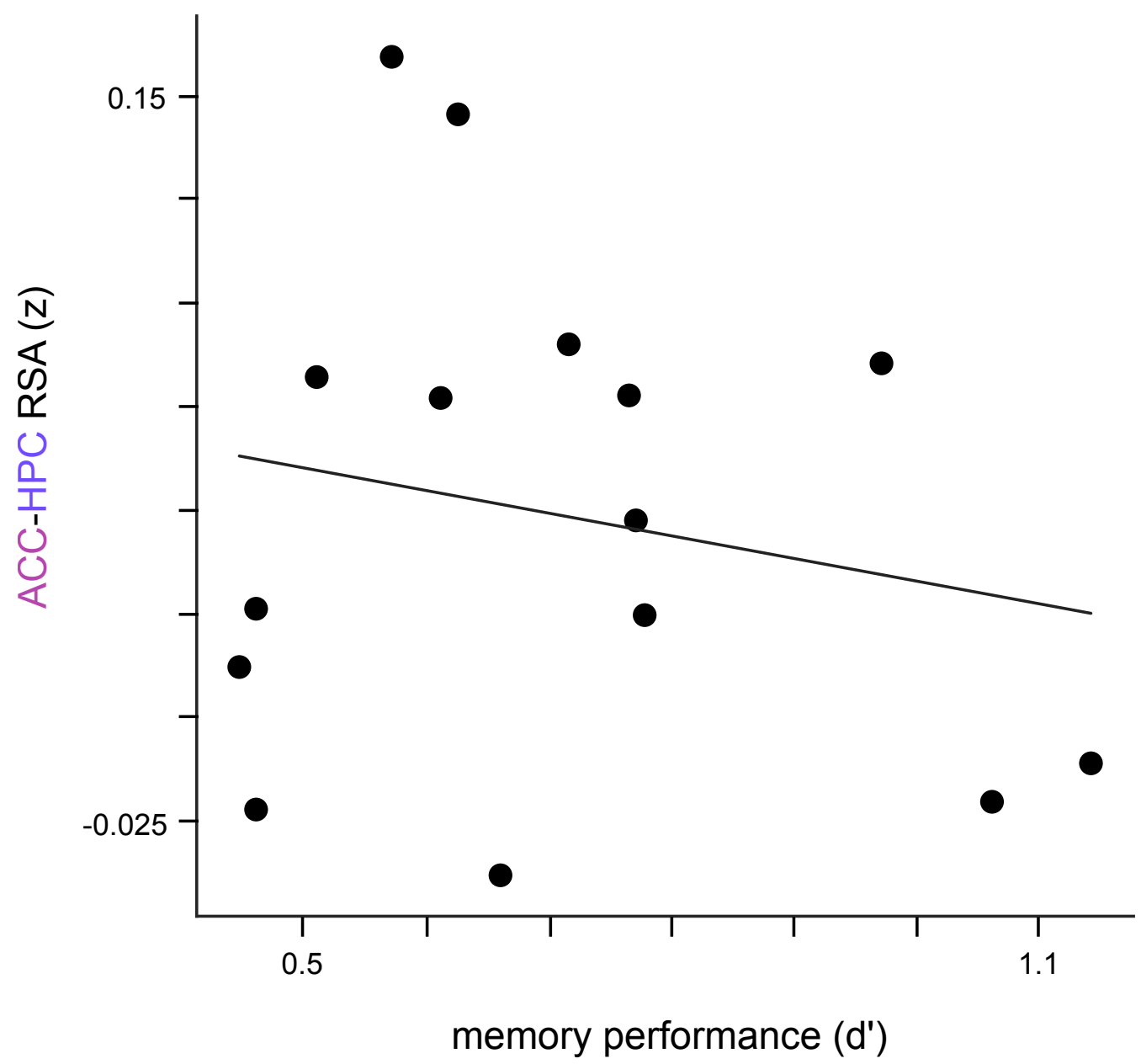

Figure S10: **HPC reinstatement of ACC feedback activity not explained by memory performance.** Scatter plots of the relationship between ACC-HPC RSA and memory performance.

bioRxiv preprint doi: https://doi.org/10.64898/2026.09.10.750374; this version posted September 12, 2026. The copyright holder for this preprint (which was not certified by peer review) is the author/funder, who has granted bioRxiv a license to display the preprint in perpetuity. It is made available under a CC-BY 4.0 International license.

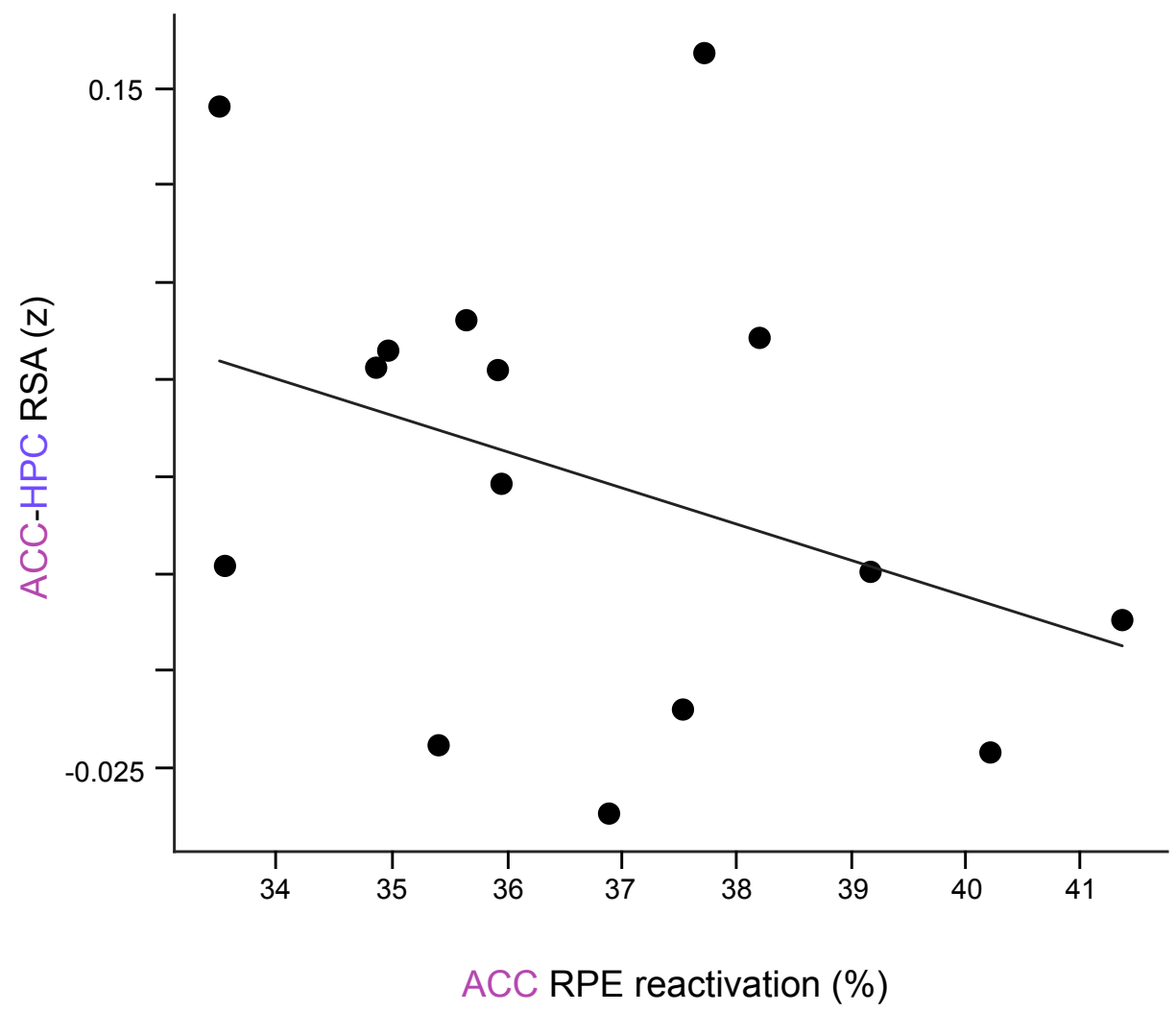

Figure S11: **HPC reinstatement of ACC feedback activity not explained by ACC RPE reactivation.** Scatter plot of the relationship between ACC reactivation in the significant cluster and ACC-HPC RSA in the significant cluster.

bioRxiv preprint doi: https://doi.org/10.64898/2026.09.10.750374; this version posted September 12, 2026. The copyright holder for this preprint (which was not certified by peer review) is the author/funder, who has granted bioRxiv a license to display the preprint in perpetuity. It is made available under a CC-BY 4.0 International license.

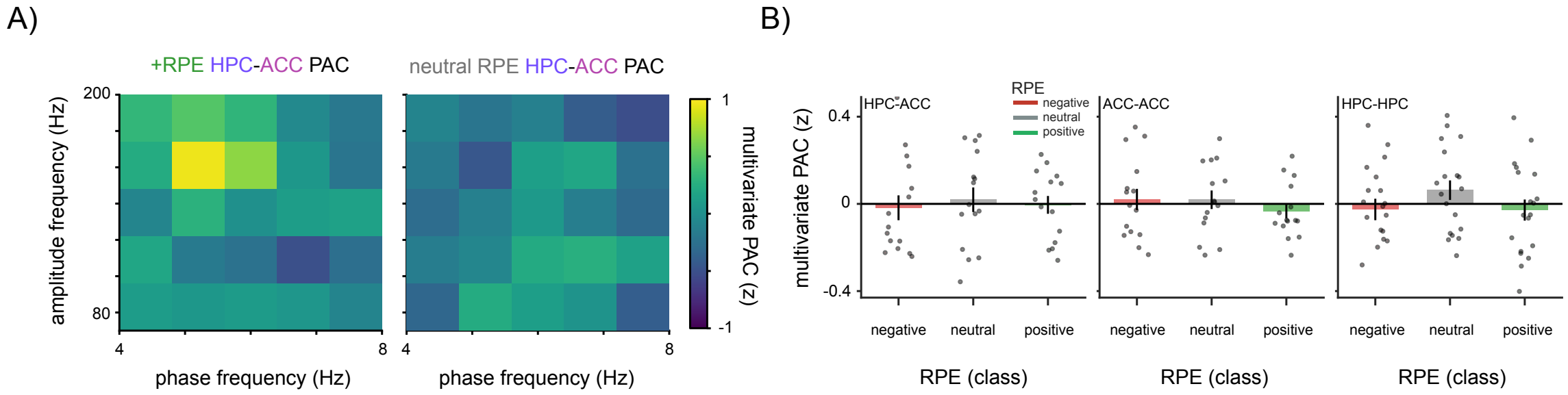

Figure S12: **No evidence that RPE induces coupling between theta phase and HFA amplitude in or between regions.** A) Example of multivariate phase-amplitude coupling computed between hippocampal theta phase and ACC HFA amplitude during the recognition cue period for positive RPE images (left) and neutral RPE images (right). Brighter colors denote larger z-scored phase-amplitude coupling. B) Mean phase-amplitude coupling during the cue period for negative, neutral, and positive RPE images for HPC-ACC (left), ACC-ACC (middle), and HPC-HPC (right). Bar height denotes mean across subjects (dots).

bioRxiv preprint doi: https://doi.org/10.64898/2026.09.10.750374; this version posted September 12, 2026. The copyright holder for this preprint (which was not certified by peer review) is the author/funder, who has granted bioRxiv a license to display the preprint in perpetuity. It is made available under a CC-BY 4.0 International license.

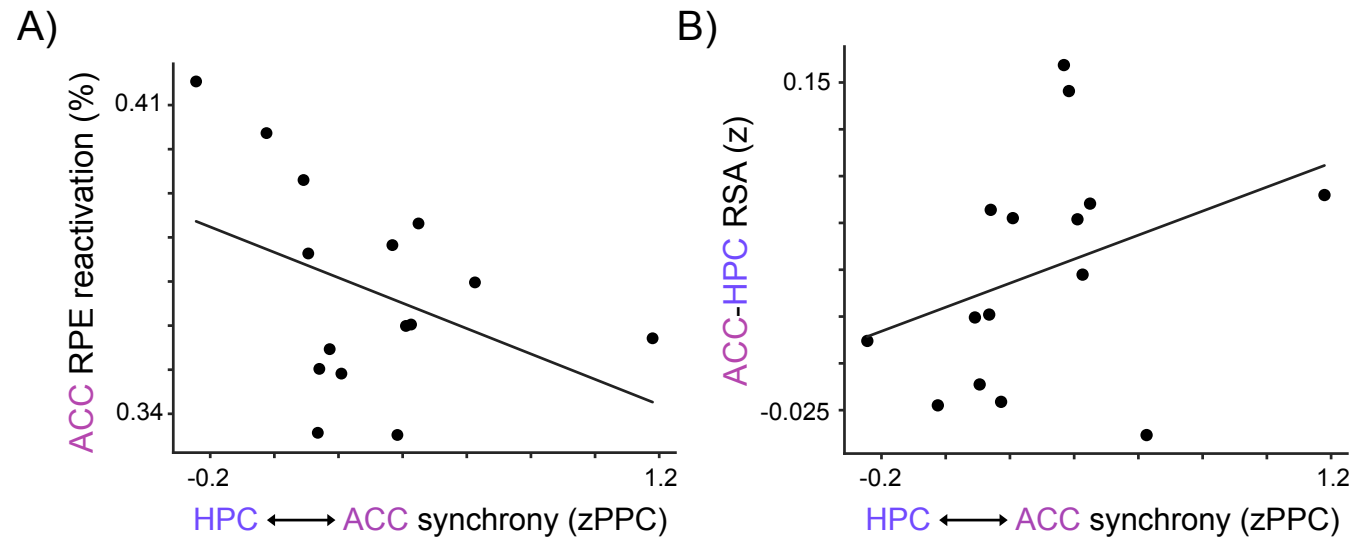

Figure S13: **ACC reinstatement and HPC reinstatement of ACC feedback activity not explained by HPC-ACC synchrony.** B) Scatter plot of the relationship between HPC-ACC theta synchrony during positive RPE cue trials and ACC reinstatement in the significant cluster. B) Scatter plot of the relationship between HPC-ACC theta synchrony during positive RPE cue trials and ACC-HPC RSA in the significant cluster.
